# Development and Evaluation of Chitosan Nanoparticles for Ocular Delivery of Tedizolid Phosphate

**DOI:** 10.3390/molecules27072326

**Published:** 2022-04-04

**Authors:** Mohd Abul Kalam, Muzaffar Iqbal, Abdullah Alshememry, Musaed Alkholief, Aws Alshamsan

**Affiliations:** 1 Nanobiotechnology Unit, Department of Pharmaceutics, College of Pharmacy, King Saud University, Riyadh 11451, Saudi Arabia; makalam@ksu.edu.sa (M.A.K.); aalshememry@ksu.edu.sa (A.A.); malkholief@ksu.edu.sa (M.A.); 2 Department of Pharmaceutics, College of Pharmacy, King Saud University, Riyadh 11451, Saudi Arabia; 3 Department of Pharmaceutical Chemistry, College of Pharmacy, King Saud University, Riyadh 11451, Saudi Arabia; muziqbal@ksu.edu.sa; 4 Bioavailability Unit, Central Lab, College of Pharmacy, King Saud University, Riyadh 11451, Saudi Arabia

**Keywords:** tedizolid-phosphate, chitosan, nanoparticles, antibacterial, eye-irritation transcorneal-permeation

## Abstract

This study investigates the development of topically applied non-invasive chitosan-nanoparticles (CSNPs) for ocular delivery of tedizolid phosphate (TZP) for the treatment of MRSA-related ocular and orbital infections. An ionic-gelation method was used to prepare TZP-encapsulated CSNPs using tripolyphosphate-sodium (TPP) as cross-linker. Particle characterization was performed by the DLS technique (Zeta-Sizer), structural morphology was observed by SEM. The drug encapsulation and loading were determined by the indirect method. In-vitro release was conducted through dialysis bags in simulated tear fluid (pH 7) with 0.25% Tween-80. Physicochemical characterizations were performed for ocular suitability of CSNPS. An antimicrobial assay was conducted on different strains of Gram-positive bacteria. Eye-irritation from CSNPs was checked in rabbits. Transcorneal flux and apparent permeability of TZP from CSNPs was estimated through excised rabbit cornea. Ionic interaction between the anionic and cationic functional groups of TPP and CS, respectively, resulted in the formation of CSNPs at varying weight ratios of CS/TPP with magnetic stirring (700 rpm) for 4 h. The CS/TPP weight ratio of 3.11:1 with 10 mg of TZP resulted in optimal-sized CSNPs (129.13 nm) with high encapsulation (82%) and better drug loading (7%). Release profiles indicated 82% of the drug was released from the TZP aqueous suspension (TZP-AqS) within 1 h, while it took 12 h from F2 to release 78% of the drug. Sustained release of TZP from F2 was confirmed by applying different release kinetics models. Linearity in the profile (suggested by Higuchi’s model) indicated the sustained release property CSNPs. F2 has shown significantly increased (*p* < 0.05) antibacterial activity against some Gram-positive strains including one MRSA strain (SA-6538). F2 exhibited a 2.4-fold increased transcorneal flux and apparent permeation of TZP as compared to TZP-AqS, indicating the better corneal retention. No sign or symptoms of discomfort in the rabbits’ eyes were noted during the irritation test with F2 and blank CSNPs, indicating the non-irritant property of the TZP-CSNPs. Thus, the TZP-loaded CSNPs have strong potential for topical use in the treatment of ocular MRSA infections and related inflammatory conditions.

## 1. Introduction

**Figure 1:**
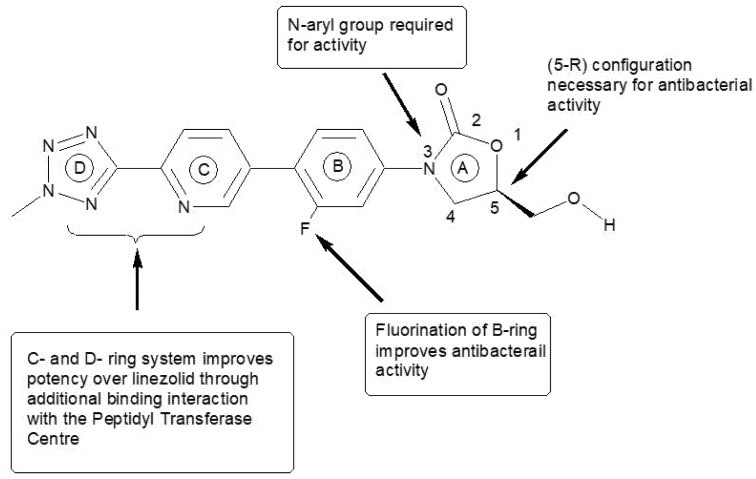
Structure–activity relationships for tedizolid. Where, A–D symbolize the different aromatic rings in the molecular structure of Tedizolid. The Ring-A = Oxazolidinone ring, Ring-B = Aryl group, Ring-C = meta-fluorine and para-oriented electron withdrawing or unsaturated ring and Ring-D = para-oriented ring structure, provides additional sites for H-bonding.

Among ocular infections, methicillin-resistant *Staphylococcus aureus* (MRSA) infections in the eyes and orbits are the most important. Such infections are often treated inappropriately [1]. The most common presentations of ocular infections by MRSA are keratitis (36%), eyelid problems (24%), conjunctivitis, cellulitis, and dacryocystitis (20%) and around nearly half (48%) of the infections are found to be vision-threatening [2]. As per the Kaiser Permanente study, roughly 13% of ocular MRSA infections were found in infants, where conjunctivitis was the main sign [3]. Due to many serious infections caused by MRSA, it has become a significant clinical challenge and economic burden [4]. Tedizolid phosphate (TZP) is a novel oxazolidinone antibiotic to treat the infections caused by MRSA that has become a new defense weapon [5]. It is also used against the vancomycin-resistant *enterococci* [6] and some linezolid-resistant strains [7]. It was approved by the US-FDA in June 2014 for acute bacterial skin and skin structure infections [8]. The chemical structure and structural activity relationships of tedizolid (TDZ) are illustrated in Figure 1. TZP is a prodrug which is rapidly converted in vivo to its active form TDZ by acid and alkaline phosphatases [9,10]. Therefore, either TDZ or TZP can be used in eye preparations. It differs from other members of the oxazolidinone class as it has a modified side chain at the C5 position of the oxazolidinone nucleus which instructs the activity against some linezolid-resistant microorganisms and has an optimized C- and D-ring system that improve its potency through additional binding site interactions [8]. The antibacterial activity of TZP/TDZ is facilitated by inhibiting the bacterial protein synthesis. Linezolid is also an oxazolidinone antibiotic approved by the FDA in 2000; however, it induces peripheral and optic neuropathy in humans, so its clinical use is limited for prolonged therapy, while TZP/TDZ has no such adverse effects [9,11].

Although vancomycin is the choice of antibiotic for the treatment of MRSA-infections, its efficacy has been compromised due to emergence of resistant strains of *S. aureus* [12].

These finding encouraged us to develop a topically applied non-invasive nano-carrier for ocular delivery of TZP to treat MRSA-related eye and orbital infections. We presumed that TZP would stand a better chance of accomplishing the critical prerequisite for new antibiotics in this era of increasing multi-drug resistance, including MRSA and other resistant strain eye infections. After topical administration, the ocular availability of drugs is limited due to strong self-protective and defensive ocular barriers. The nasolacrimal drainage, noncorneal absorption, and robust corneal impenetrability [13] limit the ocular availability (5–7%) of topically applied drugs [14,15,16]. The availability of drugs can be improved by prolonging the precorneal retention of the dosage forms and enhancing the corneal and conjunctival transport of the drugs. In some conditions repeated application of dosage forms into eyes is needed which may cause corneal pigmentation, mechanical injury, or sensitivities to the eyes [17]. To avoid the frequent application of eye preparations and to attain an effective and prolonged drug concentration into ocular tissues, the development of an appropriate dosage form is needed. Drug encapsulation into nano-carriers is one of the best approaches to overcome the shortfalls of conventional ophthalmic dosage forms [17,18,19]. Such carriers extend the ocular retention of the drug which can improve its transcorneal flux and intraocular availability [20,21].

Chitosan (CS) is hydrophilic, mucoadhesive, non-toxic, biodegradable polysaccharide [22,23], which also stabilizes tear fluids and increases the precorneal/corneal contact time of CSNPs [24]. Due to high viscosity and sufficient adhesion with the ocular surfaces CSNPs may reduce nasolacrimal drainage [25,26] and consequently, improve the ocular bioavailability of encapsulated TZP [27] which will augment its activity against Gram-positive and MRSA infections with reduced dosing frequency and easy topical instillation with good patient compliance.

Thus, we developed and characterized CS based nanoparticles (NPs) to prolong ocular retention and achieve an effective drug concentration. For the in vitro release of TZP, physicochemical characterization of a TZP-CSNP suspension for ocular suitability was performed. Antibacterial activity of TZP from NPs was determined against *B. subtilis* and *S. aureus* strains including one MRSA strain (SA-6538). Transcorneal permeation of TZP from CSNPs was tested in excised rabbit corneas and eye irritation from CSNPs was tested in rabbit eyes [28,29]. In vivo efficacy of TZP-CSNPs was estimated by analyzing the aqueous humor concentration of tedizolid (active form of TZP), which was reported in our previous publication [30].

## 2. Materials and Methods

### 2.1. Materials

Tedizolid phosphate (C_17_H_15_FN_6_O_6_P; MW 450.318 Da) was of ≥98% purity, purchased from “Beijing Mesochem Technology Co. Ltd. (Beijing, China)”. Low MW Chitosan (50–190 kDa) based on viscosity 20–300 cP, at 1 wt.% in acetic acid (1%) at 25 °C and 75–85% de-acetylated, Tripolyphosphate-sodium (TPP) and sodium dihydrogen phosphate (KH_2_PO_4_) were purchased from Sigma-Aldrich (St. Louis, MO, USA). Glacial acetic acid, HPLC grade methanol and acetonitrile were purchased from BDH, Ltd. (Poole, UK). RC-dialysis membrane (MWCO: 12–14 kDa) was purchased from Spectra Por, Spectrum Laboratories Inc., (Rancho Dominguez, CA, USA). Mannitol was purchased from Qualikems Fine Chem Pvt. Ltd. (Vadodara, India). Purified water was obtained using a Milli-Q^®^ water purifier (Millipore, Molsheim, France). All other chemicals used were of analytical grade and solvents of HPLC grade.

### 2.2. Chromatographic Analysis of TZP

Reverse-phase (RP) high-performance liquid chromatography (HPLC) with UV-detection (at 251 nm) was used for the quantification of TZP following the reported HPLC-UV method [31,32]. In brief, an HPLC system (Waters^®^ 1500-series controller, Milford, MA, USA) was used, which was equipped with a UV-detector (Waters^®^ 2489, dual absorbance detector, Milford, MA, USA), a binary pump (Waters^®^ 1525, Milford, MA, USA), and an automated sampling system (Waters^®^ 2707 Autosampler, Milford, MA, USA). The HPLC system was monitored by Breeze software. An RP C_18_ analytical column (Macherey-Nagel 250 × 4.6 mm, 5 μm) at 40 °C was used for this analysis. The mobile phase consisted of 65:35 *v*/*v* of 0.02 M sodium acetate buffer (the pH was adjusted to 3.5 by hydrochloric acid) and acetonitrile was pumped isocratically at 1 mL/min of flow rate. The total run time was 10 min. The injection volume was 30 µL. The standard stock solution of TZP was prepared in methanol (100 μg·mL^−1^) and working standard solutions (0.25–50 μg·mL^−1^) were prepared by serial dilution of the stock solution with 65:35, *v*/*v* mixture of the mobile phase.

### 2.3. Formulation Development

The TZP-loaded CSNPs were prepared by ionic-gelation of chitosan (CS) with a cross-linker of tripolyphosphate-sodium (TPP) [33], with slight modification for highly lipophilic drugs [22,23,34,35]. Briefly, 10 mg of TZP was dissolved in 200 µL of DMSO in triplicate. The drug solution was added slowly (with magnetic stirring at 500 rpm) into a previously prepared 13.5 mL 0.4, 0.6, and 0.8% *w*/*v*, solutions of CS in 1%, *v*/*v* glacial acetic acid (pH 3.0). Simultaneously, the TPP solutions in Milli-Q water (at 0.2, 0.4 and 0.6%, *w*/*v*) were prepared and pH of these solutions was maintained at 7.2 with 100 mM potassium dihydrogen phosphate buffer. Thereafter, 6.5 mL of TPP solution was added dropwise (at the rate of 1.5 mL·min^−1^) to 13.5 mL of CS solution containing TZP with continuous magnetic stirring at 700 rpm for 4 h at 10 °C [36]. The details of the constituents used to prepare three optimal TZP-loaded CSNPs are summarized in Table 1. The excess drug (possibly un-encapsulated) was washed by centrifugation (13,500 rpm) for 15 min at 10 °C. Finally, collection of TZP-loaded CSNPs was performed by washing with Milli-Q^®^ water through ultracentrifugation (30,000 rpm) for 30 min at 4 °C. Around 10 mL of CSNP suspension was filtered through a 450 μ filtration unit, frozen at −80 °C, freeze-dried (at −50 °C and 0.01 mbar pressure for 24 h) in a FreeZone-4.5 freeze dry system (Labconco Corporation, MO, USA), and stored at −20 °C for further studies. Mannitol (1%, *w*/*v*) was added into the suspension as cryoprotectant before freeze-drying [37].

### 2.4. Characterization of the CSNPs

#### 2.4.1. Particle Size, Polydispersity-Index (PDI) and Zeta-Potential Measurements

The hydrodynamic diameter as particle size, polydispersity-index (PDI) and zeta potentials of the developed CSNPs were evaluated by dynamic light scattering (DLS) analysis using a Zetasizer Nano Series (Nano-ZS, Malvern Instruments Ltd., Worcestershire, UK) [38]. The DLS also known as photon correlation spectroscopy, measures the Brownian movement and relates this to particle’s size by enlightening the particles with the laser and analyzing the fluctuations in the intensities of the scattered light, then utilizes this to calculate the particle’s size. DLS was performed at a fixed detection arrangement of 90° angle to the laser light and the center of the cuvette area. The suspensions of CSNPs were further diluted with Milli-Q^®^ water for the above measurements, because low a concentration of samples is beneficial for maximizing the amount of scattering from the measurement sample. For zeta potential, by considering the dielectric constant of water (≈78.5) at 25 °C, the electrophoretic mobility was determined and then the Henry equation was applied (these processes were performed by the software, DTS V-4.1, Malvern, UK). The magnitude of zeta potential (mV) gives an indication of the potential stability of any colloidal system. All the measurements were performed in triplicate.

#### 2.4.2. Transmission Electron Microscopy (TEM)

The morphology and structural characterization of the optimal formulation (TZP-CSNPs, F2) was carried out using transmission electron microscopy (TEM), JEOL TEM (JEM-1010). The TEM analysis was performed under light microscopy, operated at 80 kV with point-to-point resolution [39]. The magnification of images was 50–80 K (X). A combination of bright-field imaging at increasing magnification and diffraction modes was used to expose the structure and size of the NPs. The suspension of F2 was further diluted with Milli-Q water prior to the analysis. Dilution was performed to overcome certain challenges including the images overlapping, difficulty in detection of small particles, and obscured signals during observation due to the presence of the surrounding matrix and background noise. In order for the electron beams to transmit through a very thin specimen and interact with it, a drop of the nanosuspension was put on the carbon coated copper grids and stained with Phosphotungstic acid (2% solution). The grids were air dried overnight and then the particle morphology was observed at ambient temperature.

#### 2.4.3. X-ray Diffraction Study

The X-ray diffraction study on powdered samples was performed using an Ultima-IV Goniometer (Rigaku, Inc., Tokyo, Japan) over a 5.0° to 70.0° 2*θ* range at a scan speed of 1.0° per min to examine the crystalline nature of the encapsulated drug into the CSNPs as compared to the pure drug. The X-ray tube anode material was Cu with K_a_2 elimination, the K_a_2/K_a_1 intensity ratio was 0.10 nm, and it was monochromatized with graphite crystal. The diffractograms were obtained at 40 kV tube voltage and 40 mA, and the generator was in step scan mode (step size 0.02° and counting time was 1 s per step).

#### 2.4.4. Encapsulation Efficiency and Drug Loading Capacity

The encapsulation and loading of TZP into the CSNPs were determined by indirect methods (i.e., quantification of unencapsulated drugs). The amount of TZP encapsulated into NPs and the percentage drug loading were calculated by the difference between the total (initial) amounts of drug used for the preparation of the NPs and the drug analyzed in the supernatant after centrifugation of the suspension of CSNPs [10]. Briefly, 4 mg of CSNPs was suspended in methanol, vortexed and centrifuged at 13,500 rpm for 15 min. Supernatant was collected and the concentration of drug in the supernatant was analyzed by HPLC-UV [31,32]. The percentages of encapsulation efficiency (%EE) and drug loading (%DL) were calculated by Equations (1) and (2)*:*

(1) $$\%\;EE\;=\left(\frac{Initial\;amount\;of\;TZP\;used\;\left(\mathrm{mg}\right)-Amount\;of\;TZP\;in\;supernatant\;{\left(\mathrm{mg}\right)}_{\;}}{Initial\;amount\;of\;TZP\;used\;\left(\mathrm{mg}\right)\;\;}\;\right)\times 100$$

(2) $$\%\;DL=\left(\frac{Initial\;amount\;of\;TZP\;used\;\left(\mathrm{mg}\right)-Amount\;of\;TZP\;in\;supernatant\;{\left(\mathrm{mg}\right)}_{\;}}{Total\;amount\;of\;CSNPs\;\left(\mathrm{mg}\right)}\;\right)\times 100\;$$

#### 2.4.5. Physicochemical Characterization

The physicochemical characterization of TZP-loaded CSNPs was performed to ensure its suitability for ocular use. The characterization parameters included the transparency of the nanosuspension of TZP-CSNPs by visual observation under light alternatively against black and white background at 25 °C and pH 7.2. The drug content in the TZP-CSNPs was estimated by the HPLC-UV method as described above. The pH of the CSNP suspension was measured using a calibrated pH meter (Mettler Toledo MP-220, Schweiz, Switzerland) and osmolarity was checked using an Osmometer (Fiske Associates, Waterford, PA, USA). The viscosity of the CSNP suspension was determined at ocular physiological (≈35 ± 0.5 °C) and non-physiological (≈25 ± 0.5 °C) temperatures [40] using a sine-wave vibro viscometer (Model SV-10, A & D Co., Ltd., Tokyo, Japan). The viscosity of simulated tear fluid (STF) was also measured as a control for comparative analysis.

### 2.5. In Vitro Drug Release and Release Kinetics

The suspension of optimal formulation (F2) was made isotonic with mannitol solution and subjected to in vitro drug release study. Simulated tear fluid (STF) with 0.25%, *w*/*v* of Tween-80 was used as a release medium for this experiment. The STF was prepared by dissolving NaCl (3.4 g), NaHCO_3_ (1.1 g), KCl (0.7 g), and CaCl_2_·2H_2_O (0.04 g) in 500 mL of Milli-Q^®^ water. A dialysis bag was used as a release barrier [41]. Around 1 mL of F2 suspension (~821.5 µg of TZP) was put into the dialysis bags, and both ends of the bags were tied with threads. The bags filled with formulation were put into beakers containing 50 mL of STF. All the beakers were put into a shaking water bath (100 strokes per min) at 37 ± 1 °C. At different elapsed times, 1 mL aliquots were taken out from the beakers and an equal volume of fresh release medium was put into the beakers after each sampling. The collected aliquots were centrifuged at 13,500 rpm (10 min at 10 °C). The supernatants were collected and 30 µL was injected into the HPLC-UV system to analyze the TZP concentration. The drug release from TZP aqueous suspension (TZP-AqS) was also checked as a control. TZP-AqS was prepared by suspending TZP (~8.22 mg) in 10 mL of Polysorbate-20 solution (0.5%, *w*/*v*) in Milli-Q^®^ water [42,43]. All the experiments were performed in triplicate. Cumulative amount of TZP released as %DR was calculated using Equation (3).

(3) $$\%DR=\frac{Conc.\;\left(µg\cdot {\mathrm{mL}}^{-1}\right)\times Dilution\;Factor\times Volume\;of\;release\;medium\;\left(\mathrm{mL}\right)}{Initial\;dose\;of\;TZP\;used\;for\;the\;experiment\;(\mu g)}\times 100$$

In vitro release data were fitted into release kinetic model equations including zero-order, first-order, Higuchi matrix square-root, Hixson–Crowell cube-root and Korsmeyer–Peppas. The best-fit model for the release of TZP from CSNPs was classified on the basis of highest correlation coefficient (*R*^2^) value. From the slope and intercept of the plots of the kinetic models, two specific release kinetic parameters, i.e., *n* and *k* were calculated [44]. The *n*-value is also known as release/diffusion exponent, suggesting the mechanism of drug release from the CSNPs and *k* denotes the rate constant [19,45,46].

### 2.6. Antimicrobial Study

Testing of the antimicrobial activity of the F2 and TZP AqS was performed by the agar diffusion method [47,48]. Bacterial strains for the assessment were obtained from the Department of Pharmaceutics, College of Pharmacy, King Saud University. The strains were chosen from the Global Priority Pathogens List. Three Gram-positive American type culture collections (ATCC) of *Bacillus subtilis*, *Staphylococcus aureus*, and MRSA (SA-6538) were used for their TZP susceptibility (F2). The Mueller–Hinton agar (MHA) plates were prepared and each strain was spread on to the separate plates. Wells of 6 mm diameter were created by a sterile borer. In the first well, 40 µL of TZP-AqS (32.86 µg of TZP) was placed, into the second well 40 µL of F2 (~32.86 µg of TZP), and in the third well, the same volume of blank CSNPs (without TZP) was inoculated. After 1 h, the plates were incubated at 37 °C for 24 h, and after 24 h the zone of inhibition for each product was measured. The entire assessment was performed in triplicate.

### 2.7. In Vivo Animal Study

New Zealand albino rabbits weighing 2.5–3.5 kg were made available by the College of Pharmacy, Animal care and use center, King Saud University, Riyadh, Saudi Arabia, for the in vivo eye irritation experiment. The protocol for the animal use was approved by the King Saud University Research Ethics Committee with approval number KSU-SE-18–25 (amended). Animals were housed in light-controlled air-conditioned areas at 75 ± 5% RH according to the Guide for the Care and Use of Laboratory Animals recommended by the center. All the animals were healthy (free from any ocular clinical defects), were kept on a pellet diet (standard for rabbits) with water ad-libitum and fasted overnight before starting the experiment.

#### 2.7.1. Ocular Irritation Study

Based on the performance of physical and physicochemical characteristics, in vitro drug release, only the optimal formulation (F2) was chosen for the eye irritation test, which was compared with the blank formulation. The irritation study was performed by following Draize’s test in healthy rabbits [29]. The study was performed following the guidelines of the Association for Research in Vision and Ophthalmology (ARVO) for animal use in ophthalmic and vision research. According to these guidelines, only one eye (the right eye) of all rabbits was chosen for the test formulations and 0.9% NaCl was put into the left eyes (as negative control) to assess the ocular safety of the products. Normally, for one test formulation, a maximum of six rabbits is used. In the present study, we used only three rabbits for one test formulation, as we expected there might be some severe eye irritation and ocular damage, as suggested in a previous report [49]. Thus, six rabbits were divided in two groups for the irritation test of F2 and blank CSNPs (without TZP). Around 40 μL of each product was put into the lower conjunctival sac of each animal of the respective groups. All the rabbits received three consecutive doses in the conjunctival sac of right eyes at intervals of 10 min for the acute eye irritation test. After 1 h of exposure, the treated eyes were periodically examined for any injuries or signs and symptoms in the iris, cornea, and conjunctiva, or any alteration in the treated eyes as compared to the normal eyes. The photographs were captured by slit lamp microscope (Model-4ZL, Takagi, Japan) for irritation scoring purposes. The level of eye irritation was evaluated according to the guideline for scoring [28] on the basis of discomfort to the animals as well as the signs and symptoms such as swelling, redness, edema, or chemosis in the cornea, conjunctiva, and iris or any watery/mucoidal discharge [50]. The scoring was performed and the irritation potential of the tested formulations was categorized according to the described systems [51,52].

#### 2.7.2. Transcorneal Permeation

In vitro transcorneal permeation of TZP from CSNPs (F2) across the rabbit cornea, was performed using double-jacketed transdermal diffusion cells assembled with the automated sampling system SFDC 6, LOGAN, New Jersey, NJ, USA [50]. The rabbits used in the irritation test were kept on a washout period for three weeks. After injecting an overdose of a mixture of Ketamine, HCl, and Xylazine, the animals were sacrificed. Eyes were taken out and the corneas were separated. The freshly excised cornea (permeation barrier) was fitted between the donor and receptor compartments in such a way that the epithelial layer of the cornea faced towards the donor compartment of the cell. The receptor compartment of the diffusion cells was filled with STF (pH 7.4) containing Tween-80 (0.25%, *w*/*v*). A small magnetic bar was also put into the receptor compartment. The cells were placed on the LOGAN instrument and water at 37 ± 1 °C, was run through the outer jacket. For each group (in triplicate), 500 μL of suspensions of F2 (~410.8 µg of TZP) and TZP-AqS (410.8 µg of TZP) were placed in the donor compartments and the instrument was started with magnetic stirring. Samples from the receptor compartment were collected at different time points up to 4 h. The continuous magnetic stirring could remove air bubbles (if generated during sampling) from the receptor compartment. The concentration of the drug (µg·mL^−1^) that had passed through the cornea and present in the collected samples was analyzed by the HPLC-UV method [31,32]. The amount of drug that had permeated across the cornea was calculated by considering the volume of receptor compartment (5.2 mL), the cross sectional area (0.5024 cm^2^) and the initial concentration of TZP (*C_0_* = 821.6 µg·mL^−1^) using Equation (4) and plotted against time.

(4) $$Amount\;of\;drug\;permeated\;\left(µg\cdot {\mathrm{cm}}^{-2}\right)=\frac{Conc.\;\left(µg\cdot {\mathrm{mL}}^{-1}\right)\times DF\times Volume\;of\;receptor\;compartment\;\left(\mathrm{mL}\right)}{Area\;of\;cornea\;involved\;({\mathrm{cm}}^{2})}$$

The slope of this plot was used to determine the permeation parameters (steady-state flux, *J*, and apparent permeability, *P_app_*). The *P_app_* is also known as the permeation coefficient. These permeation parameters were calculated using Equations (5) and (6).

(5) $$J\;\left({µ\mathrm{gcm}}^{-2}\cdot h^{-1}\right)=\frac{dQ}{dt}$$

(6) $$P_{app}\;\left(\mathrm{cm}\cdot h^{-1}\right)=\frac{J}{C_{0}}$$

where *Q* is the amount of TZP crossed through the cornea, (*^dQ^/_dt_*) is the linear ascent of the slope, *t* is the contact time of the product with the epithelial layer of corneal, and *C*_0_ is the initial drug concentration present in the donor compartment of the diffusion cell.

### 2.8. Statistical Analysis of the Data

The data are presented as mean with standard deviation (±SD) unless otherwise indicated. Statistical analysis was performed using GraphPad Prism: Version 5 (GraphPad Software, Inc., San Diego, CA, USA). The parameters were compared by *t*-test with *p* values less than 0.05 (*p* < 0.05) considered statistically significant.

## 3. Results and Discussion

### 3.1. Formulation Development

The ionic-gelation method was used for preparation of the CSNPs where TPP sodium acted as cross-linker [33]. The TZP-CSNPs were optimized by considering the excipients (CS and TPP) concentrations and keeping 120 min of stirring time. The optimization of TZP-CSNPs was performed following our previous publication wherein we optimized indomethacin-loaded CSNPs using a three-factor three-level Box–Behnken experimental design [34]. Thus, in the present study, optimal concentrations of CS and TPP (0.6 and 0.4 mg/mL, respectively) with 120 min stirring time and 10 mg of TZP, resulted in CSNPs with the desired features. Constraints, including the minimum particle size with maximum encapsulation efficiency (%EE), drug loading (%DL) and zeta potential (ZP), were applied for optimization of the TZP-CSNPs. Based on the obtained responses (parameters mentioned in Table 2), the F2 CSNPs were found to be the best one among the three formulations tried (F1–F3). Thus, this formula was selected for further study.

The ionic interaction between the high charge density (six ionic groups) of negatively charged functional groups of TPP and the positively charged quaternary amine groups (NH_4_) of CS resulted in optimal CSNP formation at particular weight ratios of CS/TPP, with magnetic stirring at 700 rpm at room temperature. Among the three (F1–F3) formulations, F2 was chosen for further studies, based on its smallest particle size with maximum encapsulation efficiency and comparatively better loading capacity. Briefly, at low weight ratio of CS/TPP (81:26 mg with 10 mg of TZP) and at magnetic stirring rate of 700 rpm for 3–4 h was found suitable to obtain optimum-sized particles (129.13 nm) with high encapsulation (82.15%) and better drug loading capacity (7.02%), as shown in Table 2. Before putting the drug into CS solution, it was dissolved in 200 µL of DMSO, due to the highly lipophilic and poorly soluble nature of TZP. It was only 1%, (*v*/*v*) of total volume of the formulation, which is permissible because even for in vitro cytotoxicity studies. In general, by increasing the CS concentration, particle size increases; however, in the case of F2, the size was smaller than F1. This might be due to the fact that the CS was exactly 3.12-fold higher than TPP in F2, while it was 4.15-fold in case of F1. The size of F3 was unexpectedly high, which might be due to very low weight ratio of CS/TPP (CS was 2.77-fold TPP), therefore, due to lack of proper weight ratio of CS/TPP, the ionic interaction between them could not occur properly. Contrary to F1, in F2, the anionic functional groups of TPP showed better ionic interaction with the positively charged amine groups of CS due to their proper weight ratio, which might be the reason for its improved physical performance.

Chitosan (CS) was chosen as main excipient to develop the TZP-loaded CSNPs, because of its natural hydrophilic, biodegradable, and mucoadhesive properties with a non-toxic and non-irritant (to eyes) safety profile. It was expected that CS would maintain and stabilize tear fluids on ocular surfaces, hence would reduce the drainage and prolong the ocular contact time of the nanocarrier [14,19,53]. It has shown minor broad-spectrum antibacterial activity [54] against some Gram-positive and Gram-negative bacteria and also has some antifungal activity [55,56,57].

Moreover, CS was found to sustain the intraocular penetration of loaded drugs by binding with corneal epithelium and causing reversible loosening of tight junctions of corneal epithelium. Hence, it was determined to be one of the best natural polymers (of biological origin) for ophthalmic purposes [58]. It has been extensively utilized for the development of numerous products for ocular use including nanoemulsions [59], indomethacin-loaded nanocapsules [33], cyclosporine-A-loaded CSNPs [60], ofloxacin [27], and acyclovir-loaded microspheres [61]. Moreover, due to electrostatic interaction with the negatively charged mucin layers, the corneal and conjunctival epithelial penetration of CS-NP/liposome-CSNP complexes were achieved [28,61].

### 3.2. Particle Characterization and Morphology of CSNPs

Dynamic light scattering (DLS) analysis by Zetasizer was used for the characterization of the developed CSNPs including the size, polydispersity, and zeta potential. In the case of F1, at CS/TPP (54 mg and 13 mg each) and 10 mg TZP, the obtained particle size (227.23 nm) was larger, with a higher PDI value (0.833). The zeta potential was +20.6 mV and encapsulation efficiency was comparatively lower (61.4%), but the loading capacity was similar (7.97%), as compared to F2. F2, with CS/TPP weight ratio of 81/39 mg and 10 mg of drug, the obtained particle size was largest (129.13 nm), with a PDI of 0.373. For F3, a CS/TPP weight ratio of 108/39 mg and 10 mg of drug, the obtained particle size was the largest of the three formulations (472.06 nm) with a slightly higher PDI (0.576), as compared to F2 (0.373). The resultant low particle size of the developed CSNPs in this investigation could be suitable for ocular application as human eyes can tolerate the particulate materials with sizes ≤10 μ without any potential ocular irritation or corneal abrasion while the larger particles may cause scratching of ocular surfaces and discomfort to eyes [62]. Thus, reduction in nanoparticle size would improve patient compliance and provide comfort during the dose administration.

**Figure 2:**
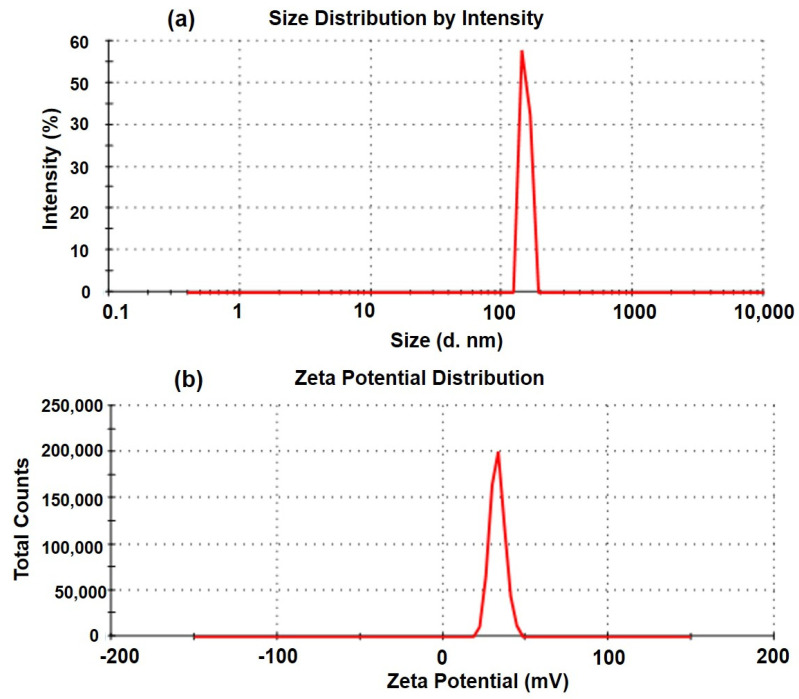
Particle size distribution (**a**) and zeta potential distribution (**b**) of the optimized TZP-loaded CSNPs (F2).

For F3, the zeta potential was +36.6 mV which is excellent for stable dispersion of the CSNPs but the encapsulation efficiency (69.92%) and drug loading (4.45%) were the lowest among the three (F1–F3) developed formulations. The results of physical characterization, including the particle size, PDI, zeta potential, encapsulation efficiency (%EE), and drug loading (%DL), are summarized in Table 2. The high positive zeta potential values of CSNPs (+20.6 to +36.6 mV) obtained in the present investigation, predict good physical stability of the developed colloidal nanocarriers (CSNPs). The same surface charges (positive) have strong electrostatic repulsion among the NPs to prevent self-aggregation. The polydispersity index measures the NPs’ size distribution, where small values are indicative of the unimodal distribution and stable dispersion of the CSNPs in the medium. The particle size and zeta potential distribution curves of the optimized CSNPs are represented in Figure 2a,b, respectively.

**Figure 3:**
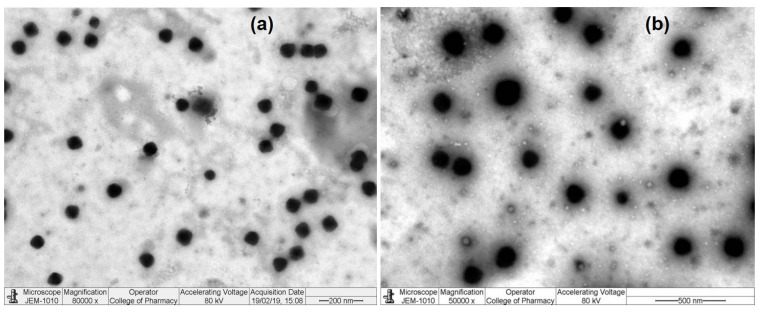
TEM images of TPZ-loaded CSNPs (F2): Performed at 80,000 magnification and 200 nm scale (**a**) and at 50,000 magnification and 500 nm scale (**b**).

Therefore, based on the above findings (Table 2), F2 was selected as the best formulation among the three developed formulations (F1–F3). To substantiate its suitability for ocular application, F2 was chosen for morphological characterization by TEM imaging. The TEM imaging of F2 revealed discrete spherical particles, well separated from each other (i.e., without potential aggregation) with solid, densely structured NPs. TEM images at two separate magnifications (80 K and 50 K) are shown in Figure 3.

The characterization of NPs involves the exploration of the structures at the nano scale. The size, shape, and any surface layers/absorbents on NPs is a crucial first step to understand the relationships between NPs, performance, quality, and safety/toxicity. It is also important whether any changes have occurred as a result of sample preparation, e.g., oxidation/reduction, during the process of checking the morphology of NPs by TEM [39,63]. Due to some challenges, such as image overlapping, difficulty in detection of small particles, and obscured signals during observation due to the presence of the surrounding matrix and background noise, the samples were diluted with Milli-Q water before the analysis. This enabled the electron beams to transmit through the highly diluted specimens and interact with them for surface imaging, when the NPs should be present around the vacuum to be free of any interference [64]. Thus, vacuum and the voltage of the electron-beam irradiation are important conditions because the highly dispersed NPs remain mobile under the electron-beam irradiation, which may interfere with the imaging. Therefore, the TEM analysis was performed under light microscopy operated at 80 kV accelerating voltage to provide high resolution and prevent any damage caused by higher-energy electron irradiation. The low accelerating voltage (80 kV), as compared to higher energy (200–1000 kV) electrons used for metallic particles and intermediate voltage (200–400 kV) for high resolution electron microscopy of non-metallic and biological specimens.

### 3.3. X-ray Diffraction Analysis

**Figure 4:**
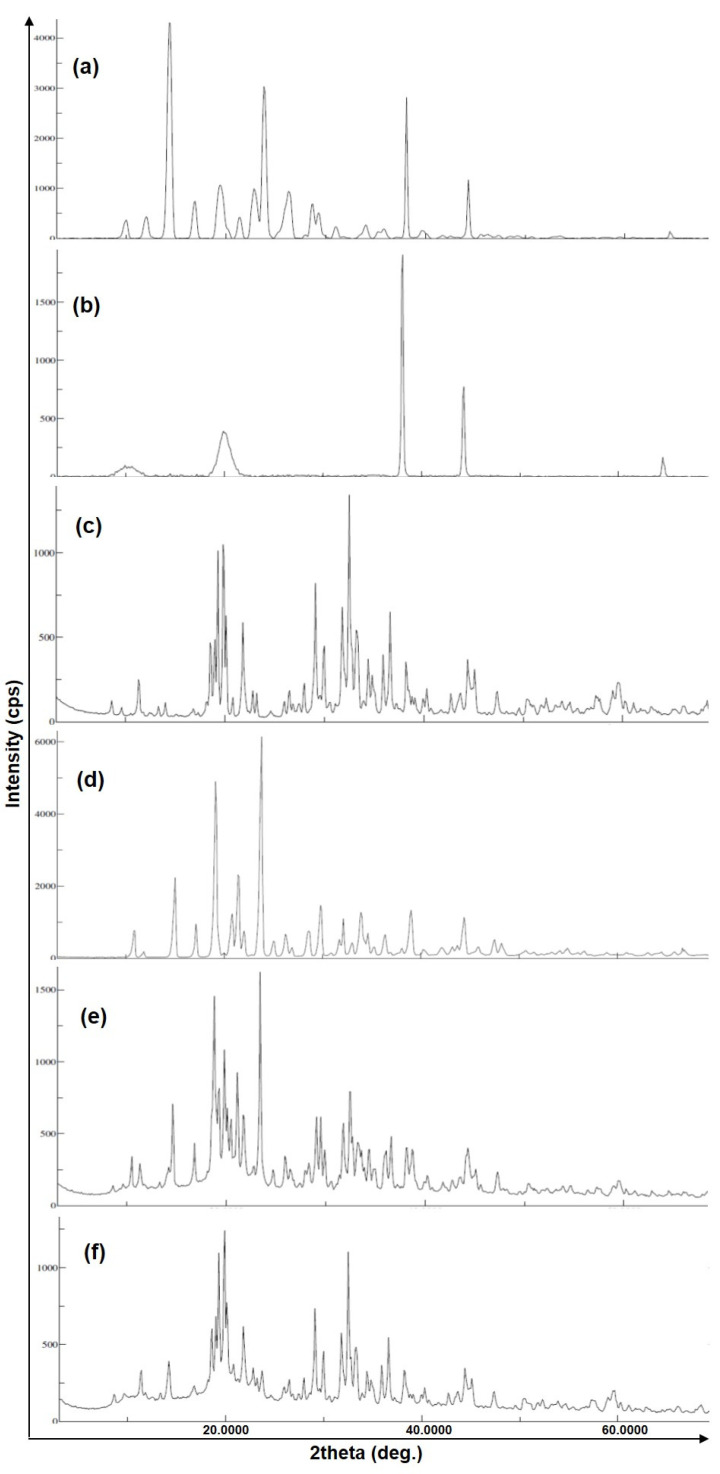
XRD patterns of pure TZP (**a**), low MW CS (**b**), TPP (**c**), mannitol (**d**), TZP-CSNPs (F2) lyophilized with mannitol (**e**), and TZP-CSNPs (F2) (**f**).

The X-ray diffractogram spectra of TZP, pure TZP, low molecular weight chitosan (CS), Tripolyphosphate sodium (TPP), mannitol, and TZP-loaded CSNPs (F2) are illustrated in Figure 4. The diffractogram of pure TZP (Figure 4a) has characteristic sharp and intense peaks at 2*θ* values of 14.4°, 23.8°, 38.1°, and 44.3°, with intensities of 3490 cps (with I/I_0_ of 100 and Bragg’s or *d*-value 6.145), 2526 cps (with I/I_0_ of 73 and *d*-value 3.735), 2492 cps (with I/I_0_ of 72 and *d*-value 2.36) and 1036 cps (with I/I_0_ of 30 and *d*-value 2.04), respectively, indicating the crystallinity of pure TZP. The diffractogram of low-molecular-weight CS (Figure 4b) has only two intense peaks at 2*θ* values of 38.0° and 44.2° with intensities of 1524 cps (I/I_0_ of 100 and *d*-value 2.366) and 593 cps (I/I_0_ of 39 and *d*-value 2.047), while the presence of a less intense (237 cps) broad peak at 2*θ* of 19.9° with a *d*-value of 4.457 and I/I_0_ of only 16.0, suggests the less crystalline, or somewhat amorphous, characteristics of CS. Figure 4c (for TPP), shows intense peaks at 2*θ*s of 19.8°, 29.1°, 32.5°, and 36.6°, with intensities of 696 cps (I/I_0_ of 79 and *d*-value 4.48), 564 cps (I/I_0_ of 64 and *d*-value 3.066), 884 cps (I/I_0_ of 100 and *d*-value 2.753), and 468 cps (I/I_0_ of 53 and *d*-value 2.453), respectively, suggesting the crystallinity of TPP. Figure 4d (for mannitol) has intense peaks at 2*θ* values of 15.0°, 19.1°, 21.4°, and 23.8° with intensities of 1814 cps (I/I_0_ of 36 and *d*-value 5.901), 4026 cps (I/I_0_ of 80 and *d*-value 4.642), 1861 cps (I/I_0_ of 37 and *d*-value 4.148), and 5092 cps (I/I_0_ of 100 and *d*-value 3.736), indicating the crystalline character of mannitol.

The diffractogram of TZP-encapsulated CSNPs (F2) lyophilized with mannitol (Figure 4e) has low intensity characteristic peaks of TZP at 2*θ* values of 14.6°, 23.4°, 38.7°, and 44.3°, with intensities of 366 cps (I/I_0_ of 36 and *d*-value 6.062), 1042 cps (I/I_0_ of 100 and *d*-value 3.798), 213 cps (I/I_0_ of 21 and *d*-value 2.324), and 203 cps (I/I_0_ of 20 and *d*-value 2.043), indicating that the TZP was entrapped in the core of the NPs or in the matrix of the polymer in an amorphous state and there was no any degradation interaction with the mannitol. Similarly, in Figure 4f, almost diminished or very low intensity characteristic peaks of TZP can be seen. However, the characteristic peaks of CS at 2*θ*s of 19.3° and 38.2° with intensities of 543 cps (I/I_0_ of 77 and *d*-value 4.595) and 169 cps (I/I_0_ of 169 and *d*-value 2.453) can be seen in Figure 4f. Moreover, the characteristic crystalline peaks of TPP at 2*θ*s of 32.5° and 36.6° with intensities of 713 cps (I/I_0_ of 100 and *d*-value 2.752) and 342 cps (I/I_0_ of 48 and *d*-value 2.453), indicate that the TZP was well encapsulated in amorphous form into the core of CSNPs rather than adsorbed onto the surfaces of the NPs.

### 3.4. Physicochemical Characterization

The transparency, drug content, osmolarity, pH, and viscosity of the TPZ-CSNPS were tested and are summarized in Table 3. Osmolarity of the CSNPs was measured in the range of 302–306 mOsmol·L^−1^, which is almost equal to the osmolarity of tear fluid (302 mOsmol·L^−1^) in normal eye conditions [65]. The viscosity of F2 (20.85 cPs at 35 °C, normal ocular surface temperature) was almost equal to the optimum viscosity (20 cPs) that the human eye can easily tolerate without any blurring of vision.

### 3.5. In Vitro Drug Release and Kinetics

**Figure 5:**
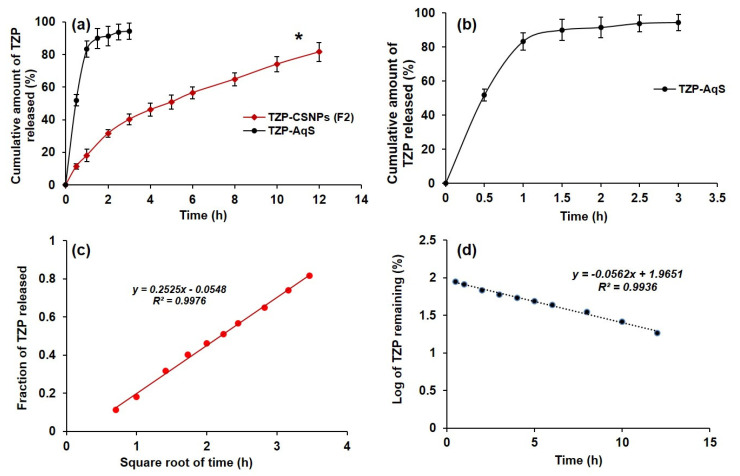
In-vitro release profile of TZP in STF from CSNPs as compared to TZP-AqS (**a**); from the aqueous suspension only (**b**); release kinetics of TZP from CSNPs (F2) that followed Higuchi’s square root of time plot (**c**); and second-best fit was the first-order model (**d**). “*” *p* < 0.05; TZP-CSNPs (F2) vs. TZP-AqS.

The in vitro release of the drug through dialysis bags in simulated tear fluid (pH 7) with 0.25% *w*/*v* of Tween-80 was found to be suitable for the release of TZP from NPs and the aqueous suspension. Tween-80 was added to increase the solubility of the highly lipophilic and poorly soluble nature of TZP into aqueous environment. The in vitro drug release profile (Figure 5a,b) shows that around 82% of the drug was released from the TZP-AqS within 1 h, while it took 12 h to release 78% of the drug from the NPs in a sustained manner. From assessment of the release profiles, TZP-AqS showed that almost all the drug was released from the suspension within 3 h, suggesting that the optimized TZP-loaded CSNPs (F2) could be an important tool for prolonged and sustained release of TZP for topical ocular application.

The sustained release property of the NPs was further confirmed by applying the release kinetics models [17]. In general, the CSNPs show a two-step release pattern—an initial burst release phase followed by a slow-release pattern. In the present investigation only sustained release of the drug occurred from the CSNPs, which might be due to the low aqueous solubility of the drug. This is also beneficial to maintain the therapeutic index of the drug for prolonged effect with reduced dosing frequency.

Applying the different kinetic models, it was observed that the in vitro release of TZP from F2 could be better explained by two models (Higuchi’s square root and first order release models). The curve between the square root of time and the fraction of drug released was almost linear (Higuchi’s square root model) and its extrapolation crossed through the origin (Figure 5c). The linearity in the release profile (as suggested in Higuchi’s square root model) indicated the sustained release property of the optimized CSNPs (F2). Among the applied models, the highest value of the correlation coefficient (*R*^2^), 0.9976, was found with the Higuchi’s square root model (Table 4). Considering the *R*^2^ values and slope of different kinetic equations, the diffusion or release exponent (*n*-value) was calculated. The obtained *n*-value (0.109) according to Higuchi’s square root model (for F2) indicated that the mechanism of drug release from F2 followed Fickian diffusion. Apart from Higuchi’s square root model, the second-best fit model for the release of TZP from the optimized CSNPs was the first-order model (with *R*^2^ = 0.9936) (Figure 5d). The values of the correlation coefficient and release-exponents are presented in Table 4.

In general, the sustained release of drugs from the biodegradable polymeric matrix (CS-matrix in the present study) is assumed to occur by three different mechanisms—(a) release of drug from the polymer matrix due to the erosion of the matrix, (b) diffusion of drug molecules through the polymer matrix, or (c) a combination of diffusion of drug molecules and degradation of polymer matrix [19,66,67]. The pattern of drug release from CSNPs in the present investigation is indicative of the mechanism of degradation and erosion of chitosan molecules, which was the reason for the continuous, sustained release of TZP from F2 and control of the release pattern for up to 12 h.

### 3.6. Antimicrobial Activity of TZP-CSNPs (F2)

**Figure 6:**
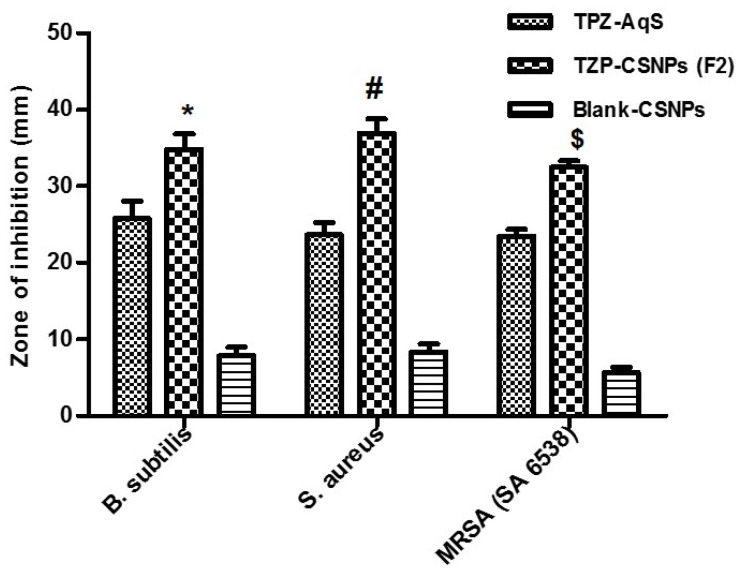
Antimicrobial activity of TZP-containing formulations as compared to blank CSNPs against some Gram-positive bacteria, including one MRSA strain. Results are presented as mean ± SD, *n* = 3. “*” *p* < 0.05; F2 vs. other formulations (for *B. subtilis*), “#” *p* < 0.05; F2 vs. other formulations (for *S. aureus*). “$” *p* < 0.05; F2 vs. other formulations (for MRSA SA-6538).

The results of an antimicrobial susceptibility test by the agar diffusion method are summarized in Table 5A. The TZP-loaded CSNPs (F2) showed significantly (*p* < 0.05) improved activity against Gram-positive bacteria such as *B. subtilis* and *S. aureus*, including one MRSA strain (SA 6538), as compared to TZP-AqS (Figure 6). Relatively little activity was noted for the blank CSNPs, as compared to the two tested formulations.

One-way analysis of variance followed by Tukey’s multiple comparison test using GraphPad Prism V-5.0 were used to check the level of significance between the two formulations, as compared to blank CSNPs (against the tested microorganism). *p* < 0.05 was considered as the level of significance. The data obtained are presented in Table 5B. The improved antimicrobial activity of the TZP formulations indicates that the formulation processes did not alter the intrinsic or inherent antimicrobial property of TZP. Moreover, the processes did not alter the structure–activity relationship of TZP. Therefore, we can conclude that the encapsulation of TZP into the CSNPs not only increases the bioavailability of the drug but could also increase its antimicrobial potency against the tested microorganisms.

### 3.7. Ocular Irritation Study

**Figure 7:**
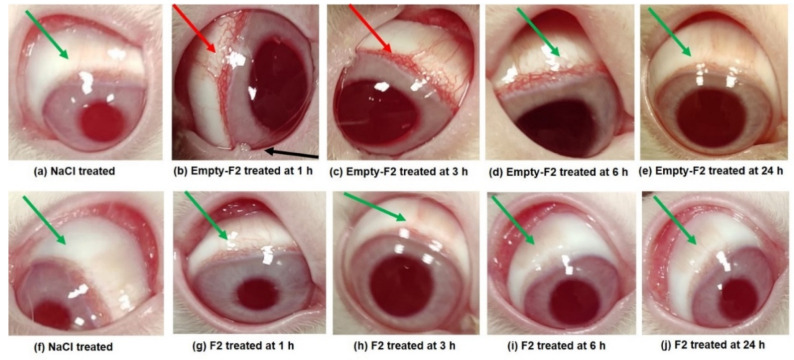
Treated rabbit eyes during irritation experiments. (**a**,**f**) showing the NaCl-treated eye of two groups. After topical application of blank CSNPs at 1 h, exhibiting mild redness (red arrow) without inflammation of conjunctiva but with mild abnormal discharge (black arrow) (**b**); at 3 h (**c**); at 6 h (**d**) and at 24 h (**e**). After topical application of TZP-CSNPs (F2) at 1 h (**g**); at 3 h (**h**); at 6 h (**i**) and at 24 h (**j**). Other images show no redness or abnormal discharge, with green arrows indicating normal features.

The scores and signs of discomfort during the eye irritation study for CSNPs (blank and F2) are shown in Table 6. No obvious symptoms of discomfort were noted in the rabbits treated with the two products. Figure 7a,f, are pictures of NaCl-, F2-, and blank CSNP- treated left eyes of rabbits, respectively. Figure 7b shows mild redness (red arrow) without inflammation of conjunctiva but with mild abnormal discharge (black arrow), 1 h after dosing with blank CSNPs. The redness and slight mucoidal discharge continued until 3 h (Figure 7c). These symptoms disappeared at 6 h (Figure 7d) and the eye regained its normal condition (green arrow) at 24 h (Figure 7e). In contrast, no such findings were noted in the F2 treated eyes even at 1 h (Figure 7g). In fact, the F2 treated eyes did not show any symptoms of irritation at any time-point (Figure 7g–j). The normal recovery in the blank CSNP-treated animals was due to the strong natural defensive mechanism of the eyes themselves. Moreover, this might be attributable to non-irritant properties of the biocompatible excipients (CS and TPP) in the formulation.

As a result of application of blank CSNPs, a slight irritation was found in one animal with some mucoidal discharge, which was given a score of 1. No opacity in the treated eyes was found. Therefore, the cornea, iris, and conjunctiva scored 0 for both the formulations. Adopting the scoring classification system for ocular irritation [52], the maximum mean total scores (MMTS) were calculated. The MMTS after 24 h, for the blank CSNPs was 19.33 (>15.1 but <25), while it was only 9.00 for F2 (>2.6 but <15) (Table 7). Therefore, the blank-CSNP formulation was judged to be “mildly irritating” while F2 was “minimally irritating” to the rabbit eyes. The low MMTS value for F2 indicates the merits of the product for ocular use.

All animals remained active and healthy throughout the study, this demonstrated that the TZP-CSNPs were non-irritant to the rabbits’ eyes. No traces of formulation were found on visual observation after 24 h, signifying the complete disposition and degradation of the treatments. Overall, the “minimally irritating” nature of TZP-CSNPs (F2) in the present investigation was demonstrated, in agreement with previous reports where chitosan based nanocarriers were applied for topical ocular delivery of dexamethasone [50], forskolin [68], and clarithromycin [69]. Thus, we conclude that TZP-loaded CSNPs were tolerated well by rabbit eyes.

### 3.8. Transcorneal Permeation of TZP

**Figure 8:**
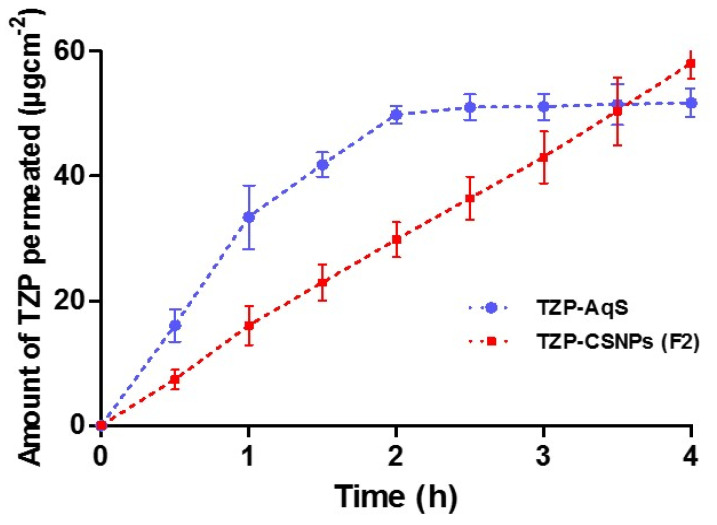
Transcorneal permeation of TZP from F2 and TZP-AqS (Mean ± SD, *n* = 3).

For this study, we used Tween-80 at 0.25%, (*w*/*v*) added to the STF to enhance the solubility of TZP into the release medium, because TZP is a highly lipophilic drug. The study was performed for 4 h only, because we did not supply any nutrients to the corneal tissue during the experiment. From the graphs in Figure 8 and the values of permeation parameters (Table 8), the CSNPs (F2) demonstrated linearity in the permeation of encapsulated drugs as compared to the conventional formulation (TZP-AqS). However, the cumulative amounts of permeated TZP at 4 h were 51.74 and 58.05 µg·cm^−2^ for TZP-AqS and F2, respectively. The pattern of drug permeation was completely different, as a significantly (*p* < 0.05) higher quantity of the drug (33.41 µg·cm^−2^) permeated from TZP-AqS within 1 h, compared to F2 (only 16.05 µg·cm^−2^ 1 h). Similarly, 49.81 µg·cm^−2^ of drug permeated from TZP-AqS at 2 h, while a similar quantity took 3.5 h from F2 (50.41 µg·cm^−2^). Around a 1.6-fold increase in flux (*J*) and *P_app_* of the drug was achieved by F2 as compared to AqS, as represented in Table 7. Finally, from the pattern of permeation profiles, we conclude that the developed nano-carriers (F2) could provide sustained delivery of the encapsulated TZP, compared to the conventional suspension of the drug. Moreover, we expected that the developed TZP-encapsulated CSNPs would enhance the prolonged and sustained release of the drug into eyes, hence would improve its ocular bioavailability.

## 4. Conclusions

The results of particle characterization, physicochemical, morphological, and in vitro release properties showed an efficient encapsulation (≈61.4–82.2%) of TZP into CSNPs by ionic gelation of CS and TPP. The reported HPLC-UV method was successful for the analysis of TZP. In vitro release profiling suggests a sustained release of TZP from optimal CSNPs (F2) for up to 12 h (81.6 ± 5.84%) in STF (pH 7) with Tween-80 (0.25% *w/v*). Release kinetics investigation on in vitro data revealed the release of TZP from F2 primarily followed the Higuchi square root model (*R*^2^ = 0.9976 and release exponent, *n* = 0.1096) indicating the mechanism was Fickian diffusion. The optimized CSNPs (F2) showed a 1.35–1.56-fold increase in the antibacterial activity of TZP against some Gram-positive microorganisms with highest value of zone of inhibition (36.9 mm) against *B. subtilis*. No sign of discomfort in the eyes of rabbits during the irritation test indicated excellent ocular tolerance, around 2–4-fold increased flux (≈28.1 µg/cm^2^/h), and apparent permeability (≈3.4 × 10^−2^ cm/h) with the highest amount of drug permeated (≈58.05 μg/cm^2^ at 4 h), indicating its higher transcorneal permeation compared to AqS.

Though it might be out of scope of the present communication, further investigation has been performed in rabbit eyes to determine the ocular bioavailability of tedizolid (the active form of TZP). Approximately 2.6 to 5.8-fold improved pharmacokinetic parameters were obtained with F2, as compared to its counter formulation (TZP-AqS). Outcomes of the investigation were reported in our previous publication during the application of a developed and validated UPLC-MS/MS method for the quantification of tedizolid in rabbit aqueous humor [30]. The CSNP-based controlled delivery of TZP would have potential ocular and other topical or oral applications. The delivery system might serve as an optimal model to encapsulate therapeutic agents including drugs, peptides, vitamins, enzymes, fatty acids, etc. Moreover, the CSNPs as carriers for TZP have strong potential for topical use treating ocular MRSA infections and associated inflammatory conditions. Further investigations are needed to validate the developed carrier system for its clinical applications to authenticate the safety and efficacy for human trials.

## 5. Future Prospects

The developed nanocarrier system (TZP-CSNPs) would be a fruitful exploration for the treatment of MRSA and other Gram-positive microbial ocular infections. This research was expected to give an excellent product at lower cost in the pharmaceutical field. This may utilize the nation’s inherent potential to provide a better platform between research (product development) and industrial collaboration. Such a collaborative approach will have all the means to achieve the ambitions, dreams, and visions of any nation. The successful achievement of the goal of this study, i.e., the encapsulation and topical ocular delivery of TZP could improve quality of life and benefit the healthcare system as follows: (a) A focus on promoting preventive care could help clinicians to reduce infectious diseases, which would encourage patients to make use of such an efficient drug delivery system as a primary step to target multiple diseases. (b) This study may help in corporatization with efficient and high-quality healthcare services and service providers that would promote competition among manufacturers and providers. This in turn would improve the capability, efficiency, and productivity of healthcare and treatment. Thus, effectively increasing the number of options available to patients. (c) To achieve the goal of corporatization, the responsibility of health care provision can be transferred to the public sector that will compete against the private sector, which will offer citizens high-quality health care facilities and allow the government to focus on its legislative, regulatory, and supervisory roles.

## Figures and Tables

**Table 1:** Formulation of tedizolid phosphate (TZP) loaded-CSNPs.

TZP-CSNPs	Amount of (mg)		
	TZP *	CS	TPP
F1	10.0	13.5 mL 0.4%, *w*/*v* (54 mg)	6.5 mL 0.2%, *w*/*v* (13 mg)
F2	10.0	13.5 mL 0.6%, *w*/*v* (81 mg)	6.5 mL 0.4%, *w*/*v* (26 mg)
F3	10.0	13.5 mL 0.8%, *w*/*v* (108 mg)	6.5 mL 0.6%, *w*/*v* (39 mg)

* In all cases the drug (TZP) was dissolved in 200 µL DMSO prior to its addition into CS solution. Low-molecular-weight chitosan (CS), tripolyphosphate sodium (TPP), nanoparticles (NPs).

**Table 2:** Physical characteristics of the TZP-CSNPs (Mean ± SD, *n* = 3).

TZP-CSNPs	Average Size (nm)	PDI	Zeta-Potential (mV)	Encapsulation Efficiency (%)	Drug Loading (%)
F1	227.23 ± 20.11	0.833 ± 0.104	+20.6 ± 0.82	61.40 ± 7.26	7.97 ± 0.94
F2	129.13 ± 21.48	0.373 ± 0.113	+31.4 ± 2.07	82.15 ± 4.08	7.02 ± 0.35
F3	472.06 ± 45.17	0.576 ± 0.093	+36.6 ± 2.06	69.92 ± 5.37	4.45 ± 0.34

F1–F3 (Formulations 1 to Formulation 3) and PDI = Polydispersity index.

**Table 3:** Physicochemical characteristics of TZP-CSNPs (mean ± SD, *n* = 3).

TZP-CHNPs	Clarity at 25 °C	Drug Content (%)	pH	Osmolarity (mOsmol·L^−1^)	Viscosity (cPs)	
					at 25 °C	at 35 °C
F1	Transparent	98.9 ± 0.4	7.5 ± 0.2	305 ± 6	21.55 ± 2.55	20.54 ± 3.17
F2	Transparent	99.5 ± 0.6	7.3 ± 0.3	302 ± 7	22.35 ±2.76	20.85 ± 2.35
F3	Transparent	98.4 ± 0.5	6.8 ± 0.9	306 ± 4	23.52 ± 2.85	21.51 ± 3.05
STF *	Transparent	…	7.4 ± 0.5	300 ± 3	01.18 ± 0.08	01.13 ± 0.07

* Simulated tear fluid (STF) was prepared by dissolving 0.68 g NaCl, 0.22 g NaHCO_3_, 0.008 g CaCl_2_·2H_2_O, and 0.14 g KCl in 100 mL of Milli-Q^®^ water, cPs (Centipoises, 1 cP = 1 mPa·s) and “…” indicates that the drug content was not measured.

**Table 4:** Release kinetics model equations.

Release Models	*R*^2^ Values	Slope	*n*-Values
Zero order (fraction of drug released vs. time)	0.9297	0.0531	0.02305
First order (log% of drug remaining vs. time)	0.9936	0.0562	0.02440
Korsmeyer–Peppas (log fraction of drug released vs. log time)	0.9848	0.5837	0.25345
Hixon–Crowell (M_o_^1/3^ − M_t_^1/3^ vs. time)	0.9798	0.0285	0.01238
Higuchi matrix (fraction of drug released vs. square root of time)	0.9976	0.2525	0.10964

*R*^2^ = Coefficient of correlation and *n* = Release/diffusion exponent.

**Table 5:** Zone of inhibitions obtained in agar diffusion test by F2 as compared to TZP-AqS. Blank CSNPs were used as control.

**(A) Microorganisms**	**Zone Diameters (mm), Mean ± SD, *n* = 3**		
	**By TPZ-AqS**	**By TPZ-CSNPs (F2)**	**By Blank CSNPs**
*B. subtilis*	25.77 ± 3.23	34.83 ± 2.78	7.83 ± 1.59
*S. aureus*	23.63 ± 2.28	36.93 ± 2.65	8.36 ± 1.47
MRSA (*SA* 6538)	23.46 ± 1.27	32.46 ± 1.18	5.66 ± 0.98
**(B) Statistical Analysis by One-Way Analysis of Variance**			
**Tukey’s Multiple Comparison Test**	**Mean Difference**	q = Sq. Root * (D/SED)	***p* < 0.05**
TZP-AqS vs. TZP-CSNPs (F2)	−10.46	10.64	Yes
TZP-AqS vs. TZP-CSNPs (F2)	17.00	17.31	Yes
TZP-AqS vs. Blank CSNPs	27.46	27.95	Yes

* SED = Standard error of the difference and D = Difference between two means, SD = Standard deviation, n = times repeated the experiment, p = probability (for significance), q = Studentized range statistic and F2 = Formulation 2 (TZP-CSNPs).

**Table 6:** Weighted irritation scores during the testing of F2 and blank CSNPs in rabbit eyes.

Lesions in the Treated Eyes	Individual Scores of Eye Irritation Experiments					
	TZP-CSNPs (F2)			Blank-CSNPs		
	Rabbit No.			Rabbit No.		
	Ist	IInd	IIIrd	Ist	IInd	IIIrd
**For Cornea**						
(A) Opacity (degree of density)	1	0	0	1	0	1
(B) Area of cornea	4	4	4	4	4	4
Total score = (A × B × 5) =	20	0	0	20	0	20
**In Iris**						
(A) Lesion values	1	0	0	1	1	0
Total score = (A × 5) =	5	0	0	5	5	0
**In Conjunctiva**						
(A) Redness	0	1	0	1	1	1
(B) Chemosis	0	0	0	0	0	0
(C) Mucoidal discharge	0	0	0	1	0	0
Total score = (A + B + C) × 2 =	0	2	0	4	2	2

F2 = Formulation 2 (TZP-CSNPs).

**Table 7:** Maximum mean total score (MMTS) calculations based on the scores represented in Table 5.

**TZP-CSNPs (F2)**					
**Rabbits**	**1st**	**2nd**	**3rd**	**SUM**	**Average (SUM/3)**
Cornea	20	0	0	20	6.67
Iris	5	0	0	5	1.67
Conjunctiva	0	2	0	2	0.66
SUM total =	25	2	0	27	9.00
**Blank-CSNPs**					
**Rabbits**	**1st**	**2nd**	**3rd**	**SUM**	**Average (SUM/3)**
Cornea	20	0	20	40	13.33
Iris	5	5	0	10	3.33
Conjunctiva	4	2	2	8	2.67
SUM total =	29	7	22	58	19.33

**Table 8:** Parameters of transcorneal permeation for F2 and TZP-AqS (Mean ± SD, *n* = 3).

Parameters	TZP-AqS	CSNPs (F2)
Cumulative amount of drug permeated (µg·cm^−2^) at 4 h	51.74 ± 2.31	58.05 ± 2.44
Steady-state flux, *J* (µg·cm^−2^·h^−1^)	17.50 ± 3.32	28.12 ± 1.41
Permeability coefficient, *P_app_* (cmh^−1^)	(2.13 ± 0.41) × 10^−2^	(3.42 ± 0.17) × 10^−2^

## Data Availability

This study did not report any data.

## Sample Availability

Samples of the compounds are available from the authors.

## References

[1] Amato M., Pershing S., Walvick M., Tanak S. Trends in methicillin-resistant staph aureus infections of the eye and orbit. Proceedings of the Annual Meeting of American Academy of Ophthalmology.

[2] Chuang C.C., Hsiao C.H., Tan H.Y., Ma D.H., Lin K.K., Chang C.J., Huang Y.C. (2012). Staphylococcus aureus ocular infection: Methicillin-resistance, clinical features, and antibiotic susceptibilities. PLoS ONE.

[3] Helzner J. (2013). Your role in curbing the rising threat of ophthalmic MRSA. Ophthalmol. Manag..

[4] Stefani S., Chung D.R., Lindsay J.A., Friedrich A.W., Kearns A.M., Westh H., Mackenzie F.M. (2012). Meticillin-resistant Staphylococcus aureus (MRSA): Global epidemiology and harmonisation of typing methods. Int. J. Antimicrob. Agents.

[5] Das D., Tulkens P.M., Mehra P., Fang E., Prokocimer P. (2014). Tedizolid Phosphate for the Management of Acute Bacterial Skin and Skin Structure Infections: Safety Summary. Clin. Infect. Dis..

[6] Ferrandez O., Urbina O., Grau S. (2016). Critical role of tedizolid in the treatment of acute bacterial skin and skin structure infections. Drug Des. Dev. Ther..

[7] Kisgen J.J., Mansour H., Unger N.R., Childs L.M. (2014). Tedizolid: A new oxazolidinone antimicrobial. Am. J. Health Syst. Pharm..

[8] Zhanel G.G., Love R., Adam H., Golden A., Zelenitsky S., Schweizer F., Gorityala B., Lagace-Wiens P.R., Rubinstein E., Walkty A. (2015). Tedizolid: A novel oxazolidinone with potent activity against multidrug-resistant gram-positive pathogens. Drugs.

[9] Schlosser M.J., Hosako H., Radovsky A., Butt M.T., Draganov D., Vija J., Oleson F. (2015). Lack of neuropathological changes in rats administered tedizolid phosphate for nine months. Antimicrob. Agents Chemother..

[10] Yang Z., Tian L., Liu J., Huang G. (2018). Construction and evaluation in vitro and in vivo of tedizolid phosphate loaded cationic liposomes. J. Liposome Res..

[11] Narita M., Tsuji B.T., Yu V.L. (2007). Linezolid-associated peripheral and optic neuropathy, lactic acidosis, and serotonin syndrome. Pharmacotherapy.

[12] Sievert D.M., Rudrik J.T., Patel J.B., McDonald L.C., Wilkins M.J., Hageman J.C. (2008). Vancomycin-resistant Staphylococcus aureus in the United States, 2002–2006. Clin. Infect. Dis..

[13] Cholkar K., Patel S.P., Vadlapudi A.D., Mitra A.K. (2013). Novel strategies for anterior segment ocular drug delivery. J. Ocul. Pharmacol. Ther..

[14] Fabiano A., Chetoni P., Zambito Y. (2015). Mucoadhesive nano-sized supramolecular assemblies for improved pre-corneal drug residence time. Drug Dev. Ind. Pharm..

[15] Fangueiro J.F., Andreani T., Fernandes L., Garcia M.L., Egea M.A., Silva A.M., Souto E.B. (2014). Physicochemical characterization of epigallocatechin gallate lipid nanoparticles (EGCG-LNs) for ocular instillation. Colloids Surf. B Biointerfaces.

[16] Zhang W., Prausnitz M.R., Edwards A. (2004). Model of transient drug diffusion across cornea. J. Control. Release.

[17] Kalam M.A., Sultana Y., Ali A., Aqil M., Mishra A.K., Chuttani K. (2010). Preparation, characterization, and evaluation of gatifloxacin loaded solid lipid nanoparticles as colloidal ocular drug delivery system. J. Drug Target..

[18] Alkholief M., Albasit H., Alhowyan A., Alshehri S., Raish M., Abul Kalam M., Alshamsan A. (2019). Employing a PLGA-TPGS based nanoparticle to improve the ocular delivery of Acyclovir. Saudi Pharm. J..

[19] Kalam M.A. (2016). Development of chitosan nanoparticles coated with hyaluronic acid for topical ocular delivery of dexamethasone. Int. J. Biol. Macromol..

[20] Akhter S., Ramazani F., Ahmad M.Z., Ahmad F.J., Rahman Z., Bhatnagar A., Storm G. (2013). Ocular pharmacoscintigraphic and aqueous humoral drug availability of ganciclovir-loaded mucoadhesive nanoparticles in rabbits. Eur. J. Nanomed..

[21] Warsi M.H., Anwar M., Garg V., Jain G.K., Talegaonkar S., Ahmad F.J., Khar R.K. (2014). Dorzolamide-loaded PLGA/vitamin E TPGS nanoparticles for glaucoma therapy: Pharmacoscintigraphy study and evaluation of extended ocular hypotensive effect in rabbits. Colloids Surf. B Biointerfaces.

[22] Kurakula M., Naveen N.R. (2020). Prospection of recent chitosan biomedical trends: Evidence from patent analysis (2009–2020). Int. J. Biol. Macromol..

[23] Kurakula M., Naveen N.R. (2020). In Situ Gel Loaded with Chitosan-Coated Simvastatin Nanoparticles: Promising Delivery for Effective Anti-Proliferative Activity against Tongue Carcinoma. Mar. Drugs.

[24] Achouri D., Alhanout K., Piccerelle P., Andrieu V.r. (2012). Recent advances in ocular drug delivery. Drug Dev. Ind. Pharm..

[25] Lihong W., Xin C., Yongxue G., Yiying B., Gang C. (2014). Thermoresponsive ophthalmic poloxamer/tween/carbopol in situ gels of a poorly water-soluble drug fluconazole: Preparation and in vitro-in vivo evaluation. Drug Dev. Ind. Pharm..

[26] Uccello-Barretta G., Nazzi S., Zambito Y., Di Colo G., Balzano F., Sansò M. (2010). Synergistic interaction between TS-polysaccharide and hyaluronic acid: Implications in the formulation of eye drops. Int. J. Pharm..

[27] Di Colo G., Zambito Y., Burgalassi S., Serafini A., Saettone M.F. (2002). Effect of chitosan on in vitro release and ocular delivery of ofloxacin from erodible inserts based on poly(ethylene oxide). Int. J. Pharm..

[28] Diebold Y., Jarrin M., Saez V., Carvalho E.L., Orea M., Calonge M., Seijo B., Alonso M.J. (2007). Ocular drug delivery by liposome-chitosan nanoparticle complexes (LCS-NP). Biomaterials.

[29] Draize J.H., Woodard G., Calvery H.O. (1944). Methods for the study of irritation and toxicity of substances applied topically to the skin and mucous membranes. J. Pharmacol. Exp. Ther..

[30] Kalam M.A., Iqbal M., Alshememry A., Alkholief M., Alshamsan A. (2021). UPLC-MS/MS assay of Tedizolid in rabbit aqueous humor: Application to ocular pharmacokinetic study. J. Chromatogr. B Anal. Technol. Biomed. Life Sci..

[31] Kennedy G., Osborn J., Flanagan S., Alsayed N., Bertolami S. (2015). Stability of Crushed Tedizolid Phosphate Tablets for Nasogastric Tube Administration. Drugs R D.

[32] Santini D., Sutherland C., Nicolau D. (2015). Development of a High Performance Liquid Chromatography Method for the Determination of Tedizolid in Human Plasma, Human Serum, Saline and Mouse Plasma. J. Chromatogr. Sep. Tech..

[33] Calvo P., Remunan Lopez C., Vila-Jato J.L., Alonso M.J. (1997). Chitosan and chitosan/ethylene oxide-propylene oxide block copolymer nanoparticles as novel carriers for proteins and vaccines. Pharm. Res..

[34] Abul Kalam M., Khan A.A., Khan S., Almalik A., Alshamsan A. (2016). Optimizing indomethacin-loaded chitosan nanoparticle size, encapsulation, and release using Boxâ€“Behnken experimental design. Int. J. Biol. Macromol..

[35] Guo H., Li F., Qiu H., Liu J., Qin S., Hou Y., Wang C. (2020). Preparation and Characterization of Chitosan Nanoparticles for Chemotherapy of Melanoma Through Enhancing Tumor Penetration. Front. Pharmacol..

[36] Almalik A., Day P.J., Tirelli N. (2013). HA-C oated Chitosan Nanoparticles for CD 44-M ediated Nucleic Acid Delivery. Macromol. Biosci..

[37] Alshememry A., Kalam M.A., Almoghrabi A., Alzahrani A., Shahid M., Khan A.A., Haque A., Ali R., Alkholief M., Binkhathlan Z. (2022). Chitosan-coated poly (lactic-co-glycolide) nanoparticles for dual delivery of doxorubicin and naringin against MCF-7 cells. J. Drug Deliv. Sci. Technol..

[38] Kurakula M., Ahmed O.A., Fahmy U.A., Ahmed T.A. (2016). Solid lipid nanoparticles for transdermal delivery of avanafil: Optimization, formulation, in-vitro and ex-vivo studies. J. Liposome Res..

[39] Rodriguez-Gonzalez V., Obregon S., Patron-Soberano O.A., Terashima C., Fujishima A. (2020). An approach to the photocatalytic mechanism in the TiO2-nanomaterials microorganism interface for the control of infectious processes. Appl. Catal. B.

[40] Qi H., Chen W., Huang C., Li L., Chen C., Li W., Wu C. (2007). Development of a poloxamer analogs/carbopol-based in situ gelling and mucoadhesive ophthalmic delivery system for puerarin. Int. J. Pharm..

[41] Eldeeb A.E., Salah S., Mabrouk M., Amer M.S., Elkasabgy N.A. (2022). Dual-Drug Delivery via Zein In Situ Forming Implants Augmented with Titanium-Doped Bioactive Glass for Bone Regeneration: Preparation, In Vitro Characterization, and In Vivo Evaluation. Pharmaceutics.

[42] Ali Y., Lehmussaari K. (2006). Industrial perspective in ocular drug delivery. Adv. Drug Deliv. Rev..

[43] Moore J. (1984). Final report on the safety assessment of polysorbates 20, 21, 40, 60, 61, 65, 80, 81, and 85. J. Am. Coll. Toxicol..

[44] Elgadir M.A., Uddin M.S., Ferdosh S., Adam A., Chowdhury A.J.K., Sarker M.Z.I. (2015). Impact of chitosan composites and chitosan nanoparticle composites on various drug delivery systems: A review. J. Food Drug Anal..

[45] Ritger P.L., Peppas N.A. (1987). A simple equation for description of solute release I. Fickian and non-fickian release from non-swellable devices in the form of slabs, spheres, cylinders or discs. J. Control. Release.

[46] Ritger P.L., Peppas N.A. (1987). A simple equation for description of solute release II. Fickian and anomalous release from swellable devices. J. Control. Release.

[47] Alangari A., Alqahtani M.S., Mateen A., Kalam M.A., Alshememry A., Ali R., Kazi M., AlGhamdi K.M., Syed R. (2022). Iron Oxide Nanoparticles: Preparation, Characterization, and Assessment of Antimicrobial and Anticancer Activity. Adsorpt. Sci. Technol..

[48] Al-Yousef H.M., Amina M., Alqahtani A.S., Alqahtani M.S., Malik A., Hatshan M.R., Siddiqui M.R.H., Khan M., Shaik M.R., Ola M.S. (2020). Pollen bee aqueous extract-based synthesis of silver nanoparticles and evaluation of their anti-cancer and anti-bacterial activities. Processes.

[49] Lee M., Hwang J.-H., Lim K.-M. (2017). Alternatives to in vivo Draize rabbit eye and skin irritation tests with a focus on 3D reconstructed human cornea-like epithelium and epidermis models. Toxicol. Res..

[50] Kalam M.A. (2016). The potential application of hyaluronic acid coated chitosan nanoparticles in ocular delivery of dexamethasone. Int. J. Biol. Macromol..

[51] Falahee K. (1981). Eye Irritation Testing: An Assessment of Methods and Guidelines for Testing Materials for Eye Irritancy.

[52] Kay J. (1962). Interpretation of eye irritation test. J. Soc. Cosmet. Chem..

[53] Hosseinnejad M., Jafari S.M. (2016). Evaluation of different factors affecting antimicrobial properties of chitosan. Int. J. Biol. Macromol..

[54] Devlieghere F., Vermeulen A., Debevere J. (2004). Chitosan: Antimicrobial activity, interactions with food components and applicability as a coating on fruit and vegetables. Food Microbiol..

[55] Badawy M.E.I., Rabea E.I., Rogge T.M., Stevens C.V., Smagghe G., Steurbaut W., Höfte M. (2004). Synthesis and Fungicidal Activity of New N, O-Acyl Chitosan Derivatives. Biomacromolecules.

[56] Harish Prashanth K.V., Tharanathan R.N. (2007). Chitin/chitosan: Modifications and their unlimited application potential—An overview. Trends Food Sci. Technol..

[57] Kanatt S.R., Rao M.S., Chawla S.P., Sharma A. (2013). Effects of chitosan coating on shelf-life of ready-to-cook meat products during chilled storage. LWT-Food Sci. Technol..

[58] Kapanigowda U.G., Nagaraja S.H., Ramaiah B., Boggarapu P.R., Subramanian R. (2015). Enhanced Trans-Corneal Permeability of Valacyclovir by Polymethacrylic Acid Copolymers Based Ocular Microspheres: In Vivo Evaluation of Estimated Pharmacokinetic/Pharmacodynamic Indices and Simulation of Aqueous Humor Drug Concentration-Time Profile. J. Pharm. Innov..

[59] Badawi A.A., El-Laithy H.M., El Qidra R.K., El Mofty H., El dally M. (2008). Chitosan based nanocarriers for indomethacin ocular delivery. Arch. Pharm. Res..

[60] De Campos A.M., Snchez A., Alonso M. (2001). Chitosan nanoparticles: A new vehicle for the improvement of the delivery of drugs to the ocular surface. Application to cyclosporin A. Int. J. Pharm..

[61] Genta I., Conti B., Perugini P., Pavanetto F., Spadaro A., Puglisi G. (1997). Bioadhesive Microspheres for Ophthalmic Administration of Acyclovir. J. Pharm. Pharmacol..

[62] Zimmer A., Kreuter J.r. (1995). Microspheres and nanoparticles used in ocular delivery systems. Adv. Drug Deliv. Rev..

[63] Datye A.K., Smith D.J.J.C.R. (1992). The study of heterogeneous catalysts by high-resolution transmission electron microscoDV. Catal. Rev..

[64] Smith D.J., Glaisher R.W., Lu P., McCartney M.J.U. (1989). Profile imaging of surfaces and surface reactions. Ultramicroscopy.

[65] Tomlinson A., Khanal S., Ramaesh K., Diaper C., McFadyen A. (2006). Tear film osmolarity: Determination of a referent for dry eye diagnosis. Investig. Ophthalmol. Vis. Sci..

[66] Nanaki S.G., Koutsidis I.A., Koutri I., Karavas E., Bikiaris D. (2012). Miscibility study of chitosan/2-hydroxyethyl starch blends and evaluation of their effectiveness as drug sustained release hydrogels. Carbohydr. Polym..

[67] Unagolla J.M., Jayasuriya A.C. (2018). Drug transport mechanisms and in vitro release kinetics of vancomycin encapsulated chitosan-alginate polyelectrolyte microparticles as a controlled drug delivery system. Eur. J. Pharm. Sci..

[68] Khan N., Ameeduzzafar, Khanna K., Bhatnagar A., Ahmad F.J., Ali A. (2018). Chitosan coated PLGA nanoparticles amplify the ocular hypotensive effect of forskolin: Statistical design, characterization and in vivo studies. Int. J. Biol. Macromol..

[69] Bin-Jumah M., Gilani S.J., Jahangir M.A., Zafar A., Alshehri S., Yasir M., Kala C., Taleuzzaman M., Imam S.S. (2020). Clarithromycin-Loaded Ocular Chitosan Nanoparticle: Formulation, Optimization, Characterization, Ocular Irritation, and Antimicrobial Activity. Int. J. Nanomed..

